# Corneal steep island formation after primary pterygium surgery

**DOI:** 10.1371/journal.pone.0313958

**Published:** 2024-11-19

**Authors:** Dong Hee Ha, Kyoung Woo Kim

**Affiliations:** Department of Ophthalmology, Chung-Ang University College of Medicine, Chung-Ang University Hospital, Seoul, Republic of Korea; Alexandria University Faculty of Medicine, EGYPT

## Abstract

**Aims:**

This study aimed to report corneal steep island (CSI) formation following primary pterygium surgery and to identify preoperative pterygium morphological features that predict the likelihood of CSI.

**Methods:**

A total of 93 eyes from 84 subjects with primary nasal pterygium, who underwent pterygium excision combined with conjunctival-limbal autograft, were included in this retrospective longitudinal cohort study. CSI was defined using anterior segment swept-source optical coherence tomography (AS SS-OCT). Eyes were divided into two groups: those with postoperative CSI formation (Group 1) and those without postoperative CSI (Group 2). We compared postoperative anterior corneal astigmatism (ACA, in diopters [D]) and root mean square (RMS) values of anterior corneal lower-order (LoA) and higher-order aberrations (HoA) between the groups. Baseline clinical severity grades of pterygium based on the pre-established pterygium body morphology and vascularity, ACA, and AS SS-OCT-guided pterygium morphological profiles (horizontal invasion length [HIL, mm], height [μm], thickness (μm), and residual corneal thickness [RCT]/central corneal thickness [CCT] ratio [RCT/CCT]) were also compared.

**Results:**

Postoperative CSI occurred in 26 eyes (28.0%) with a maximum follow-up duration of 22.9±27.4 months. Group 1 exhibited significantly higher postoperative anterior corneal RMS LoA and HoA, as well as the RMS values of the 4^th^ to 6^th^ orders. Although clinical severity grades of pterygium did not differ between groups, baseline ACA was higher in Group 1 (4.56±5.49 D vs. 2.70±3.80 D, *P* = 0.009). HIL (4.49±0.84 mm vs. 3.77±1.29 mm, *P* = 0.010) was higher in Group 1, while pterygium height (930.8±84.4 μm vs. 999.3±128.0 μm, *P* = 0.015) and RCT/CCT ratio (1.07±0.13 vs. 1.14±0.16, P = 0.049) were lower in Group 1.

**Conclusions:**

CSI may develop after primary pterygium surgery, particularly in patients with relatively higher preoperative ACA, longer HIL, and shorter height. Given that CSI can significantly increase both lower and higher-order aberrations, it is crucial to anticipate CSI probability and inform patients before surgery.

## Introduction

Pterygium is a common ocular surface condition characterized by the development of a triangular growth involving fibrous subconjunctival connective tissue and thickening of the overlying conjunctival epithelium [1]. The encroachment of the pterygial head onto the cornea and its extension towards the center of the cornea significantly change corneal curvature, mechanically flattening and distorting it in the direction of the pterygium’s traction [2, 3]. These changes can lead to significant visual impairment by inducing astigmatism, altering corneal refractive status, and increasing corneal wavefront aberrations [4].

The introduction of anterior segment swept-source optical coherence tomography (AS SS-OCT) has transformed the evaluation of anterior corneal elevation parameters in abnormal eyes [5]. Specifically, AS SS-OCT has enhanced the examination of pterygium by providing more accurate measurements of its influence on optical properties [4]. With AS SS-OCT, even subtle perioperative changes in refractive power within localized areas can be readily detected following pterygium surgery. Generally, pterygium flattens the cornea, but after its excision, the mean refractive corneal power increases as the previously flattened corneal curvature reverses post-surgery [6]. However, no study has yet revealed localized corneal refractive changes at the site of the pterygium head after surgery. It is well established that central corneal refractive surgeries can increase corneal aberrations, which may detract from visual quality [7, 8]. Although pterygium surgery principally affects the peripheral corneal surface, we hypothesized that a pterygium that invades the cornea to a degree sufficient to alter central corneal curvature may not only influence peripheral corneal refractive power but could also indirectly impact central corneal aberrations after surgery.

In this regard, the main aim of this study is to investigate the occurrence of localized refractive changes, referred to as corneal steep islands (CSIs), in subjects after undergoing primary pterygium excision. We then analyzed the impact of localized formation of CSIs on corneal optical quality, as indicated by corneal aberrations evaluated at the central 6.0 mm diameter zone using AS SS-OCT. Lastly, to anticipate the likelihood of CSI occurrence after pterygium excision, we identified preoperative refractive and morphological profiles that may serve as contributing factors.

## Materials and methods

The study was a single-center, retrospective longitudinal cohort study adhering to the principles outlined in the Declaration of Helsinki. The research protocol was approved by the Chung-Ang University Hospital Institutional Review Board (IRB) (Approval No. 2208-025-19433). Medical records of the subjects were accessed from 15 March 2023 to 9 September 2023, following IRB approval. The authors did not have access to information that could identify individual participants during or after data collection. Given the retrospective nature of the study design, the IRB waived the requirement for informed consent.

In our clinical practice, we thoroughly evaluate each pterygium patient with a standardized protocol. We utilize these pre-operative examination results to optimize clinical decisions. Consequently, the study may appear to be prospective in nature; however, it is, in fact, retrospective.

### Subjects

The study included patients diagnosed with nasal-only primary pterygium who underwent pterygium excision with conjunctival-limbal autograft between September 2021 and June 2023. Patients with medical records of AS SS-OCT (Anterion, Heidelberg, Germany) examinations performed before surgery and at least four weeks after surgery were included in the analysis. Subjects who experienced postoperative CSI during the follow-up period were designated in Group 1, while those who did not experience postoperative CSI were designated as Group 2.

### Study design

Our study design was structured as follows:

1. Identification of postoperative CSI occurrence.
2. Comparison of postoperative corneal aberrations between groups to assess the influence of postoperative CSI on corneal aberrations.
3. Comparison of baseline pterygium data between groups to identify contributing factors for postoperative CSI occurrence.

### Definition of CSI

**Fig 1:**
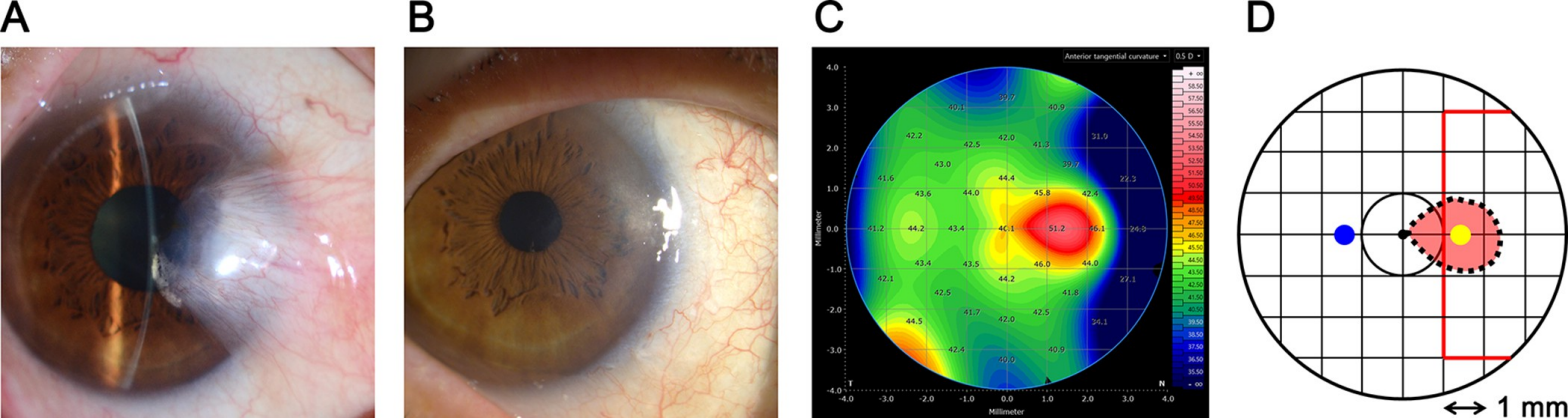
Definition of corneal steep island (CSI) occurring after pterygium surgery. (**A and B**) Anterior segment photographs before (**A**) and after primary nasal pterygium excision combined with conjunctival-limbal autograft (**B**). (**C**) The anterior tangential curvature map of the cornea after pterygium surgery from anterior segment swept-source optical coherence tomography, taken at the same time as the anterior segement photograph in **B**, with the CSI area acknowledged in a red oval. (**D**) The illustration of the topographic map shown in **C**. The CSI area is outlined with a dotted oval line. The shorter diameter of the CSI exceeded the 1.0 mm threshold required to define a CSI. The steepest point within the CSI is marked with a yellow dot, and its horizontally symmetrical point is indicated as a blue dot. The difference in K between these points was 6.59 diopters (D), exceeding the 2.0 D threshold required to define a CSI. The steepest point within the CSI was located outside of the central 1.0 mm circle but was positioned between 3.0 mm superiorly and 3.0 mm inferiorly (within the red lines nasally).

CSI was identified on the anterior tangential curvature map obtained through AS SS-OCT (Anterion), conducted at the maximum follow-up period but at least 4 weeks post-surgery, within the subject inclusion period. This determination was made according to the following three criteria (**Fig 1**):

1. The shorter diameter of the CSI area exceeds 1.0 mm.
2. The steepest point within the CSI is located nasally and between 3.0 mm superiorly and 3.0 mm inferiorly, but outside of the central 1.0 mm diameter circle.
3. The steepest K within the CSI area is at least 2.0 D higher than the K value at the horizontally symmetrical point in the same eye.

### Surgical procedures

All surgeries were performed by a single surgeon (KWK) using consistent surgical procedures. The targeted area for excision was delineated with a pen under local anesthesia. To aid in hydrodissection, a 2% lidocaine injection was administered into the conjunctival tissue, facilitating the separation of the superior epithelium from the underlying tissue. Fibrovascular tissue beneath the pterygial epithelium was dissected and removed using Westcott scissors. The pterygium head invading the cornea was carefully peeled and excised. The conjunctival-limbal autograft, harvested from the superior conjunctiva, was sized to match the excised pterygium body. After positioning the graft, it was secured onto the sclera using fibrin glue (Greenplast Q pre-filled syringe kit, GC Biopharma corporate, Yongin-si, Gyeonggi-do, Republic of Korea) and #10–0 nylon sutures.

Postoperatively, patients were instructed to apply topical moxifloxacin eye drops (Vigamox, Novartis, Basel, Switzerland) four times daily, dexamethasone and neomycin combination ointment (Maxitrol oint, Novartis) twice daily, and 20% autologous serum eye drops six times daily during the initial postoperative week, with a gradual tapering over one month. Additionally, oral prednisolone 10mg was taken once daily for five days following surgery.

### Estimation of corneal aberrations and pterygium morphological profiles based on AS SS-OCT

**Fig 2:**
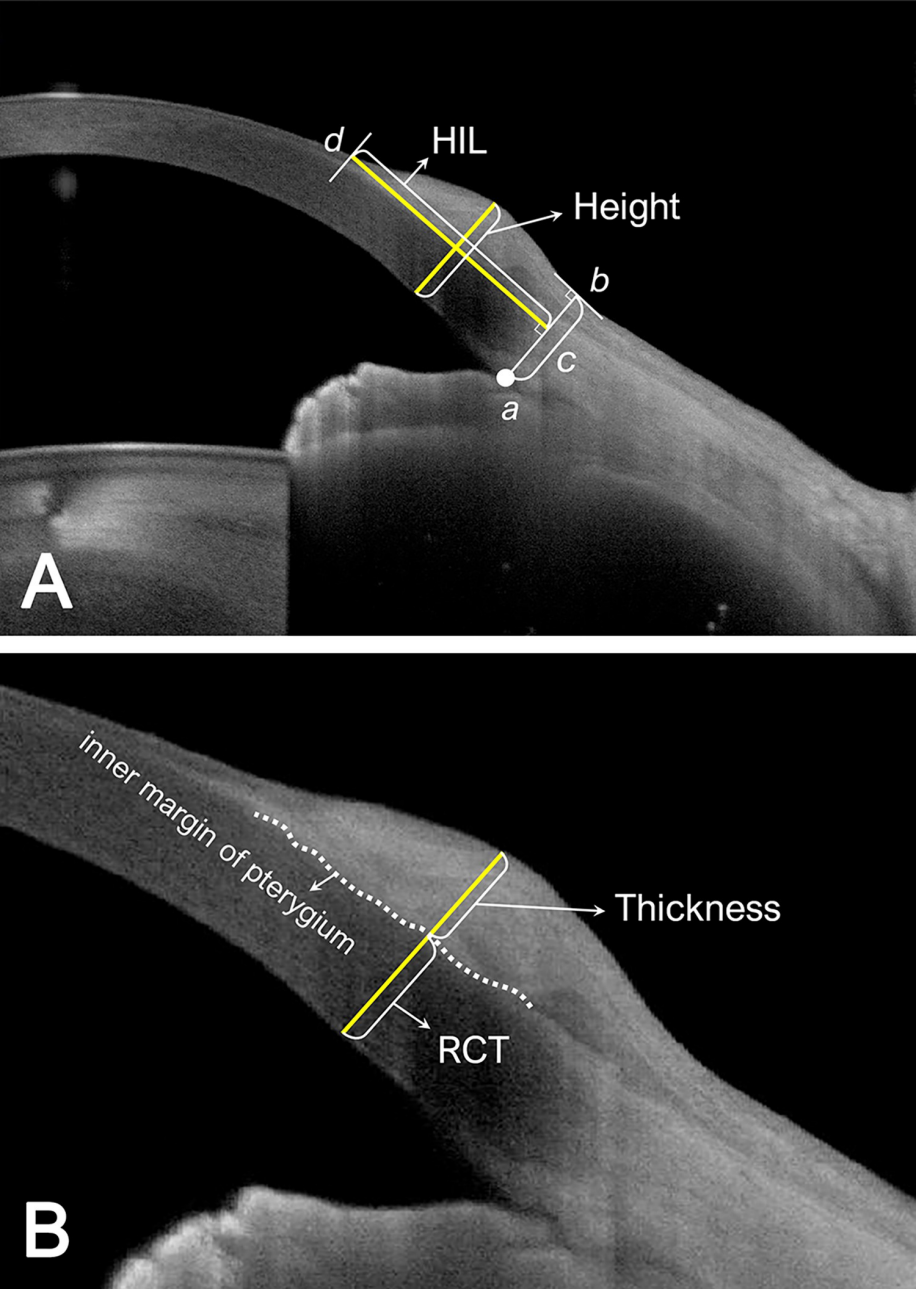
The pterygium morphological profiles observed in anterior segment swept-source optical coherence tomography. (**A**) Photograph showing the definition of horizontal invasion length (HIL) and pterygium height. HIL is described as the linear distance from the vertical line (c), which connects the anterior chamber angle (a), to a point (b) vertically extended on a tangent line to the end of the pterygium head (d). Pterygium height is defined as the vertical distance between the corneal endothelium and the outermost pterygium surface, measured at the middle of the HIL. (**B**) A magnified view of **A** to clarify the definitions of residual corneal thickness (RCT) and pterygium thickness. Pterygium thickness is outlined as the length from the inner margin of the pterygium to the outer surface within the height of the pterygium. RCT is described as the distance from the corneal endothelium to the inner margin of the pterygium within the height of the pterygium.

Measurements were acquired using AS SS-OCT (Anterion) both before and after the surgical procedure. Pre-surgery measurements were conducted within four weeks prior to the operation. Baseline data, gathered before the surgical intervention, encompassed four morphological characteristics of the pterygium, as depicted in **Fig 2**: horizontal invasion length (HIL, mm), height (μm), thickness (μm), and the ratio of residual corneal thickness in the pterygium (RCT) to central corneal thickness (CCT) (RCT/CCT). Additionally, corneal optical properties, including anterior corneal astigmatism (ACA), root mean square (RMS) lower-order aberration (LoA), and RMS higher-order aberration (HoA), were assessed.

ACA measurements were taken at a 3 mm radius from the cornea app, while RMS LoA and RMS HoA values were assessed within a 6 mm diameter zone from the cornea app following the surgery. Morphological profiles were analyzed using images from the metrics app. HIL was described as the linear distance from the vertical line connecting the anterior chamber angle (a) to a point (b) vertically extended on a surface tangent line to the end of the pterygium head (d). Pterygium height represented the vertical span between the corneal endothelium and the outermost pterygium surface measured in the middle of the HIL. Pterygium thickness indicated the length from the inner margin of the pterygium to the outer surface within its height. RCT was calculated as the distance from the corneal endothelium to the inner margin of the pterygium within its height, derived by subtracting the pterygium’s thickness from its height. CCT values were retrieved from the metrics app.

### Clinical severity grading of pterygium

All eyes affected by pterygia were assessed using three well-established grading systems to evaluate severity. These systems considered the transparency (T grade) [9] and vascularity (V grade) [10] of the pterygium body stroma, as well as the extent of morphological loss of vertical length of the plica semilunaris (LPS) [11].

In summary, the T grading system categorized pterygia into T1, T2, and T3 based on the visibility of underlying episcleral vessels. Pterygia were classified as V1, V2, and V3 according to varying levels of vascularization. The assessment of LPS was performed using digital photographs taken at a magnification of ×10 with a slit lamp imaging system while the subject was in lateral gaze, both with and without a yellow barrier filter. The extent of LPS was determined by calculating the ratio of its length within the plica semilunaris to the total length of the plica semilunaris, expressed as a percentage. This measurement followed a detailed method previously reported. Standard photographs were utilized to apply all three grading systems.

### Statistical analysis

Prism software (GraphPad, La Jolla, CA, USA) and SPSS software (Chicago, IL, USA) was used for statistical tests. To compare the data of Group 1 and Group 2, the chi-square test as well as parametric Student’s t-test or non-parametric Mann-Whitney U test, was used for analyses. To compare the baseline versus postoperative data in each group, Wilcoxon signed rank test was used. The data are presented as mean ± standard deviation (SD), and statistical significance was set at a *P* value < 0.05.

## Results

### Demographics

In this study, 93 eyes from 84 subjects diagnosed with primary nasal pterygium were included. Of the 84 subjects, 43 (51.2%) were male and 41 (48.8%) were female. The average age of all subjects was 57.4±11.6 years (standard deviation). The maximum postoperative period for AS SS-OCT evaluation was 22.6±23.0 months.

Postoperative CSI was observed in 26 eyes (28.0%, Group 1) out of a total of 93 eyes (**Table 1**). There were no differences in the maximum period for AS SS-OCT evaluation, age, and sex distribution between the groups (*P* = 0.423, *P* = 0.596, and *P* = 0.071, respectively).

**Table 1:** Demographics of subjects according to postoperative corneal steep island (CSI) occurrence.

Variables	Group		*P* value
	Group 1 (With CSI)	Group 2 (Without CSI)	
Total No. of eyes (%)	26 (28.0%)	67 (72.0%)	
Maximum period (months)^a^	22.9±27.4	22.5±21.3	0.423
Age (yrs)^b^	58.4±13.4	56.9±10.8	0.596
% Female (M:F)^c^	65.4% (10:16)	40.6% (38:26)	0.071

Data shows mean±standard deviation.

^a^Mann-Whitney U test

^b^Student’s t test

^c^Chi-square test. CSI, corneal steep island.

### The impact of postoperative CSI presence on corneal aberrations

While the occurrence of CSI was not correlated with the ACA value postoperatively, the presence of CSI was linked to an elevation in RMS values of both lower and higher anterior corneal aberrations (*P* = 0.021 and *P*<0.001, **Table 2**). The 4^th^, 5^th^, and 6^th^ order RMS values were higher in Group 1 compared to those in Group 2 (*P*<0.001).

**Table 2:** Difference of postoperative anterior corneal aberrations according to postoperative corneal steep island (CSI) occurrence.

Variables	Group		*P* value
	Group 1 (With CSI)	Group 2 (Without CSI)	
Postoperative ACA (Diopters, D)	1.09±0.55	0.92±0.67	0.118
Postoperative RMS LoA (μm)	2.72±0.98	2.28±0.58	0.021*
Postoperative RMS HoA (μm)	0.99±0.49	0.66±0.21	<0.001*
Postoperative 4^th^ order RMS (μm)	0.57±0.17	0.40±0.13	<0.001*
Postoperative 5^th^ order RMS (μm)	0.15±0.07	0.09±0.04	<0.001*
Postoperative 6^th^ order RMS (μm)	0.08±0.04	0.04±0.03	<0.001*

Data shows mean±standard deviation. Mann-Whitney U test. CSI, corneal steep island; ACA, anterior corneal astigmatism; RMS, root mean square; LoA, lower-order aberration; HoA, higher-order aberration.

*P<0.05.

### The influence of baseline clinical severity grades of pterygium on postoperative CSI occurrence

The severity grades of pterygium, based on three systems including T grade, V grade, and the extent of LPS, showed no difference between Group 1 and Group 2 (**Table 3**).

**Table 3:** Difference of clinical severity grades of pterygium according to postoperative corneal steep island (CSI) occurrence.

Variables	Group		*P* value^a^
	Group 1 (With CSI)	Group 2 (Without CSI)	
T grade^**a**^			
T1	11 (17.4%)	3 (8.3%)	0.599
T2	48 (69.6%)	18 (75.0%)	
T3	8 (13.0%)	5 (16.7%)	
V grade^**a**^			
V1	9 (14.5%)	3 (8.3%)	0.302
V2	49 (71.0%)	16 (66.7%)	
V3	9 (14.5%)	7 (25.0%)	
Extent of LPS (%)^b^	60.6±30.7	60.0±30.2	0.998

Data shows mean±standard deviation.

^a^Chi-square test

^b^Mann-Whitney U test. CSI, corneal steep island; LPS, loss of vertical length of plica semilunaris.

### Impact of baseline ACA on postoperative CSI occurrence

The preoperative ACA in Group 1 was 4.56±5.49 D, significantly higher than the 2.70±3.80 D observed in Group 2 (*P* = 0.009, **Table 4**).

**Table 4:** Difference of baseline anterior corneal astigmatism (ACA) according to postoperative corneal steep island (CSI) occurrence.

Variables	Group		*P* value
	Group 1 (With CSI)	Group 2 (Without CSI)	
Baseline ACA (Diopters, D)	4.56±5.49	2.70±3.80	0.009*

Data shows mean±standard deviation. Mann-Whitney U test. CSI, corneal steep island; ACA, anterior corneal astigmatism.

### Impact of baseline morphological profiles of pterygium on postoperative CSI occurrence

In Group 1, the HIL, height, and RCT/CCT ratio of pterygium measured 4.49±0.84 mm, 930.8±84.4 μm, and 1.07±0.13, respectively, showing significant differences compared to the measurements in Group 2, which were 3.77±1.29 mm, 999.3±128.0 μm, and 1.14±0.16, respectively (*P* = 0.010, *P* = 0.015, and *P* = 0.049, respectively) (**Table 5**). However, there were no significant differences in the thickness of pterygium value between group 1 and group 2 (*P* = 0.716).

**Table 5:** Difference of baseline morphological profiles of pterygium according to postoperative corneal steep island (CSI) occurrence.

Variables	Group		*P* value
	Group 1 (With CSI)	Group 2 (Without CSI)	
HIL (mm)^a^	4.49±0.84	3.77±1.29	0.010*
Height (μm)^a^	930.8±84.4	999.3±128.0	0.015*
Thickness (μm)^b^	364.7±63.0	388.7±116.2	0.716
RCT/CCT ratio^a^	1.07±0.13	1.14±0.16	0.049*

Data shows mean±standard deviation. ^a^Student’s t test; ^b^Mann-Whitney U test. CSI, corneal steep island; HIL, horizontal invasion length; RCT, residual corneal thickness; CCT, central corneal thickness.

## Discussion

Since pterygium primarily affects the corneal surface, previous studies have concentrated on the alterations in corneal topographic parameters induced by pterygium. It is well-documented that pterygium can result in corneal astigmatism, irregularities, and higher-order aberrations [4]. Several possible theories have been proposed to elucidate the mechanisms underlying corneal distortion and flattening caused by pterygium, including the tractional force exerted by contractile elements, localized pooling of tears at the pterygium apex, and stromal scarring [12–14]. Following pterygium excision, typical alteration involves the reduction of refractive astigmatism and topographic irregularity, as well as the steepening of the corneal curvature [15].

In this study, we present the occurrence of CSI in 28.0% of eyes with primary nasal pterygium that underwent pterygium excision combined with conjunctival-limbal autograft. The existence of postoperative CSI was significantly associated with the postoperative elevation of anterior corneal LoA and HoA and was contributed to more severe baseline corneal astigmatism and morphological features of pterygium. Since the pterygium head is located at the peripheral cornea, anatomically, its removal would primarily affect the peripheral cornea, and it would be reasonable to assume that it may not have a significant impact on visual acuity. In fact, in this study cohort, most subjects with the preoperative decreased vision experienced an improvement in visual acuity compared to preoperative levels. However, we defined CSI as being located nasally and between 3.0 mm superiorly and 3.0 mm inferiorly, although being located outside the central 1.0 mm zone. Given this definition, it is plausible that the presence of CSI influenced the increase in corneal aberrations evaluated within the central 6.0 mm diameter zone. Although visual acuity remained good, the impact of corneal LoA and HoA on optical quality may be considered secondary. Nonetheless, the association of longer HIL as a risk factor for CSI, coupled with the fact that central corneal aberrations are generally known to degrade visual quality, suggests that this study provides meaningful results indicating that delaying pterygium surgery may allow the pterygium to progress and lead to complications such as increased corneal aberrations.

Due to the significant variation in follow-up intervals among subjects, we are unable to consider postoperative CSI as an irreversible corneal change. However, unlike a previous study that revealed keratometric values, including true net power and total corneal refractive power, stabilized as soon as one week after primary pterygium excision [16], CSI was observed on average up to 22.9±27.4 months after surgery in this study. Therefore, we hypothesize that CSI may not be a temporary phenomenon. However, this hypothesis should be confirmed by prospective longitudinal long-term observation.

Generally, after pterygium excision surgery, refractive astigmatism and topographic irregularity decrease, and the cornea becomes steeper [15]. Accordingly, corneal HoA also decrease after pterygium excision, as observed using AS SS-OCT [17]. Although ACA and corneal aberrations significantly improve after surgery in both groups in this study (**S1 Table**), the postoperative values of LoA and HoA were notably different between groups, being higher in subjects with postoperative CSI occurrence.

Considering that human visual perception is significantly influenced by ocular aberrations, and specifically, HOAs negatively affect visual acuity, leading to issues such as decreased contrast sensitivity, glare, starburst, and halos [18–20], clinicians need to pay attention to the specific cohort with postoperative CSI for potential postoperative visual discomfort.

No study has yet reported the phenomenon of localized corneal steep islands (CSI) rather than generalized corneal steepening after pterygium excision [15]. Therefore, we still do not understand why CSI occurs localized on the pre-existing pterygium bed on the cornea. Intriguingly, one study evaluated corneal densitometry in pterygium and revealed that corneal densitometric values are higher in more severe pterygium cases preoperatively, and density may improve after pterygium excision surgery [21].

In this regard, we hypothesize that more severe pterygium might have been highly adapted to the relatively higher corneal density condition preoperatively. Thus, once the corneal density decreased after pterygium excision, the localized corneal stroma might be more prone to steepening compared to the pterygium with lower severity, which had lower corneal densitometric values before surgery. Similarly, in this study, the baseline pterygium morphological severities were more severe in subjects from Group 1, which indicated higher ACA, longer HIL, and shorter height, suggesting more stromal invasion vertically. On the other hand, the clinical severity grades of pterygium were not significantly different between groups. This is probably because those three grading systems are based on the pterygium body features rather than corneal invading head morphology.

Indeed, preoperative higher ACA might be a confounder in the postoperative occurrence of CSI. Unfortunately, we are not certain whether preoperative higher ACA induces CSI, nor do we understand the mechanism behind CSI occurrence after surgery, particularly in patients with higher preoperative ACA. However, we believe that our finding of significantly higher ACA in Group 1 may serve as a useful clinical predictor for CSI occurrence after pterygium surgery, based on our statistical analysis. This is because ACA can be easily measured before surgery in pterygium patients.

There are certain limitations to this study. As it is retrospective, not all subjects underwent postoperative AS SS-OCT measurements simultaneously after surgery. Additionally, there is no longitudinal analysis regarding the restoration or maintenance of the CSI. To assess the long-term outcomes of CSI, a prospective study can be conducted in the future. Future studies should include longitudinal investigations into the long-term progression or improvement of CSI to ensure clinical keratometric stability for consecutive procedures, such as refractive surgery, intraocular lens measurement for cataract surgery, and spectacle prescriptions. Moreover, factors such as patient age and pterygium duration were not assessed, as our study primarily focused on the quantitative characteristics of pterygium, evaluated through AS SS-OCT. These additional factors can be investigated in further research. Nevertheless, this study is the first to propose the occurrence of CSI after pterygium surgery and identify possible contributing factors of morphological pterygium features associated with CSI occurrence.

## Conclusion

In conclusion, a localized anterior corneal steepened area, termed CSI, may develop after primary pterygium excision combined with conjunctival-limbal autograft, particularly in patients with more severe preoperative corneal astigmatism and morphological features. Since CSI may significantly increase both lower and higher aberrations in the cornea, it is essential to predict the likelihood of CSI occurrence and inform patients in advance.

## Supporting information

S1 Table Perioperative change of anterior corneal astigmatism (ACA) and anterior corneal root mean square (RMS) aberrations according to postoperative corneal steep island (CSI) occurrence. (PDF)
