# Supplementary material for: Corneal steep island formation after primary pterygium surgery
Source: PLoS One. 2024 Nov 19;19(11):e0313958. doi: 10.1371/journal.pone.0313958 (PMC11575761; doi:10.1371/journal.pone.0313958)
Supplement: S1 Table — (PDF) [file pone.0313958.s001.pdf]

S1 Table 1. Perioperative change of anterior corneal astigmatism (ACA) and anterior corneal root mean square (RMS) aberrations according to postoperative corneal steep island (CSI) occurrence.

Variables	Group			
	Group 1 (With CSI)		Group 2 (Without CSI)	
	Baseline	Postoperative	Baseline	Postoperative
ACA (Diopters, D)	4.56±5.49	1.09±0.55	2.70±3.80	0.92±0.67
***P* value** (Baseline vs. postoperative)	<0.001*		<0.001*	
RMS LoA (μm)	6.79±4.10	2.72±0.98	4.59±3.32	2.28±0.58
***P* value** (Baseline vs. postoperative)	<0.001*		<0.001*	
RMS HoA (μm)	3.69±2.24	0.99±0.49	2.37±2.17	0.66±0.21
***P* value** (Baseline vs. postoperative)	<0.001*		<0.001*	

Data shows mean±standard deviation. Wilcoxon signed-rank test. CSI, corneal steep island; ACA, anterior corneal astigmatism; RMS, root mean square; LoA, lower-order aberration; HoA, higher-order aberration. *$P<0.05$.
